# Concurrence of novel mutations causing Gilbert’s and Dubin–Johnson syndrome with poor clinical outcomes in a Han Chinese family

**DOI:** 10.1038/s10038-022-01086-1

**Published:** 2022-10-24

**Authors:** Tai-Cheng Zhou, Xiao Li, Hui Li, Feng-Wei Liu, Si-Hang Zhang, Jing-Hua Fan, Wen-Xiu Yang, Ya-Li Yang, Liang Zhang, Jia Wei

**Affiliations:** 1 Central Lab, Liver Disease Research Center and Department of Infectious Disease, The Affiliated Hospital of Yunnan University (The Second People’s Hospital of Yunnan Province), Kunming, China; 2 Pathology Department, The Affiliated Hospital of Yunnan University (The Second People’s Hospital of Yunnan Province), Kunming, China

**Keywords:** Mutation, Genetics, Sequencing

## Abstract

Dual-hereditary jaundice (Dubin–Johnson syndrome (DJS) and Gilbert’s syndrome (GS)) is a rare clinical entity resulting from defects of the ATP binding cassette subfamily C member 2 (*ABCC2*) and UDP glucuronosyltransferase family 1 member A1 (*UGT1A1*) genes with autosomal recessive inheritance. In this study, we aimed to investigate the mutation profiles and characterize the phenotypes in a Han Chinese family with DJS and GS. Genetic screening for variants in the *ABCC2* and *UGT1A1*, immunohistochemistry for expression of ABCC2, and histopathological examination were carried out. The proband and his brother had unconjugated and conjugated hyperbilirubinemia after birth. The proband’s sister had only conjugated hyperbilirubinemia after birth. The proband developed into pleural effusions and ascites, pericardial thickening, intrahepatic and extrahepatic biliary duct dilatation, and enlarged gallbladder at age 50. Hepatocellular carcinoma occurred in the proband’s brother at age 46. Seven compound defects of the *ABCC2* gene [c.2414delG, p.(Ile1489Gly), p.(Thr1490Pro), and p.(Ile1491Gln)] and the *UGT1A1* gene (c.-3279T>G, p.(Gly71Arg), and p.(Pro451Leu)) were identified in family members. Accumulation of pigment in hepatocytes characteristic of that in DJS was present in the proband and his brother. Expression of ABCC2 protein was markedly diminished in the patient’s liver. Our results show a different genetic profile of DJS and GS in a Han Chinese family, indicating a more complex pattern of dual-hereditary jaundice among different populations. The present study illuminates the underpinnings of DJS and GS and extends the mutation profiles and phenotypes of these two syndromes in dual-hereditary jaundice.

## Introduction

Hereditary hyperbilirubinemia syndromes are caused by defects in bilirubin transport or conjugation in the liver [1]. Two genes for these syndromes are involved: UDP-glucuronosyl transferase1A1 (*UGT1A1*) and ATP binding cassette subfamily C member 2 (*ABCC2*). Defects in *UGT1A1* may impair the glucuronidation of bilirubin and may be responsible for Gilbert’s syndrome (GS; On-line Mendelian Inheritance in Man database [OMIM] No. 143500) [1, 2]. Defects in *ABCC2* are responsible for Dubin–Johnson syndrome (DJS; OMIM No. 237500) [3]. Jaundice is a common clinical presentation of both syndromes.

The prevalence of GS is high, varying from 2 to 20% among persons of various ethnicities [4]. The clinical manifestation of GS is mildly increased unconjugated bilirubin values (total serum bilirubin >20 μmol/L but usually <80 μmol/L) without hepatocellular disease or hemolysis [5]. More than 100 variants in *UGT1A1* have been reported; among them, the linked polymorphisms A (TA)_7_TAA (rs8175347) and c.-3279T>G (rs4124874) in the promoter region are most strongly associated with GS [6]. DJS is a rare disorder, characterized by conjugated hyperbilirubinemia (>7 μmol/L), melanin-like pigment deposition in hepatocytes, and normal liver function [7]. Thirty-four pathogenic variants in *ABCC2* have been identified for DJS [8].

Dual-hereditary jaundice (GS and DJS) is an atypical phenotype, described in a few studies on Europeans, that may be confused with common liver diseases. Moreover, to our knowledge, no long-term follow-up of patients with dual-hereditary jaundice has been reported, so understanding of the outcome of patients with the disease is limited. Here, we present the first reported Chinese family with dual-hereditary hyperbilirubinemia, with characterization of the molecular mechanism and clinical features of the disorder.

## Patients and methods

### Patients

All participants were members of a Han Chinese family and signed the informed consent.

### Mutational analysis

Sequences of *ABCC2* and *UGT1A1* genes were analyzed by polymerase chain reaction and direct sequencing from genomic DNA extracted from 200 µL whole blood. All exons of *ABCC2* and *UGT1A1* were amplified by polymerase chain reaction with corresponding primers [9, 10]. All sequence variants were annotated according to reference sequences (NG_011798.1 for *ABCC2* and NG_002601.2 for *UGT1A1*). Novel mutations of *ABCC2* were verified in 211 unrelated control subjects without a family history of hyperbilirubinemia.

To explore the transcriptional status of these mutations found in genomic DNA of the *ABCC2* gene, total RNA was extracted from peripheral blood mononuclear cell by using the phenol-chloroform method after TRIzol treatment (Invitrogen; 15596018). The A260/A280 ratio and concentration of total RNA was measured on a micro-spectrophotometer (NanoDrop 2000, Thermo Scientific). The integrity of RNA samples based on 28S and 18S rRNA was evaluated on a 1.5% agarose gel. cDNA was synthesized from about 500 ng total RNA by using PrimeScript^TM^ RT reagent Kit with gDNA Eraser (TaKaRa, RR047A). Exon 18 and exon 31 of the *ABCC2* gene of all family members and the full-length *ABCC2* coding sequence (CDS) of the proband were amplified from cDNA. The primers used for coding region amplification and sequencing were listed in Supplementary Table S1. All variants were recorded according to the reference sequence (NM_000392.4). TA cloning was used to verify the three consecutive heterozygous mutations in exon 31.

### Histology and immunohistochemistry

Formalin-fixed and paraffin-embedded sections of the liver were stained with hematoxylin-eosin, periodic acid Schiff (PAS), and silver ammonium complex (Masson’s method). Liver biopsy of the proband’s brother (II:9) was immunohistochemically stained for CD34, glypican, and KI-67.

### Immunohistochemistry and confocal laser scanning microscopy (CLSM)

After being deparaffinized and pretreated, microsections were double immunohistochemical stained for ABCC2 (R260, dilution1:800; Cell Signaling Technology, Farmingdale, New York, USA) and CEA-related adhesion molecule 1 (CEACAM1; clone #283324, dilution1:200; R&D systems, Minnesota, USA). Slides were next incubated with appropriate secondary antibodies conjugated to Alexa Fluor® 488 or 594 (Jackson, USA), both diluted to 1:200 with phosphate-buffered saline. Slides were mounted with Mowiol, and nuclei were stained with 4’, 6-diamidino-2-phenylindole (Boster, Wuhan, China). Slides incubated without primary/secondary antibodies were used as negative controls. Slices were processed and imaged by microscopy (Carl Zeiss AxioScope. A1) with laser at the wavelength of 350 nm for blue fluorescence (nuclei), 488 nm for green fluorescence (ABCC2), and 594 nm for red fluorescence (CEACAM1). All biopsy specimens were evaluated by two pathologists. Only strong staining in a canalicular pattern was considered positive in the immunohistochemical examination of ABCC2 and CEACAM1.

## Results

### Clinical data

**Fig. 1:**
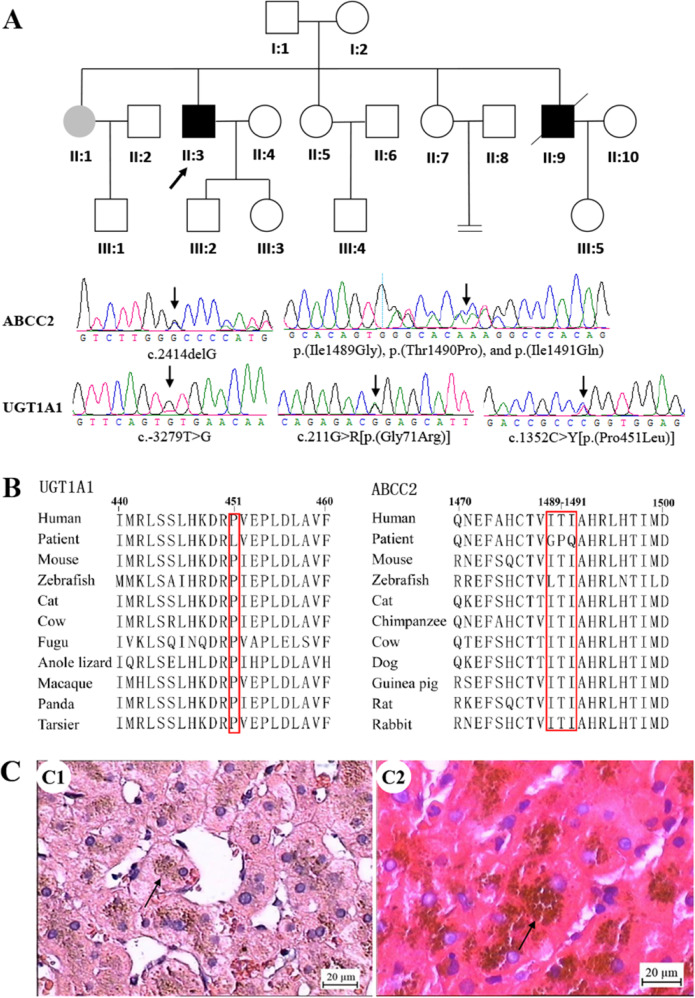
Pedigree of GS and DJS families with *ABCC2* and *UGT1A1* mutations, sequencing chromatogram, evolutionary conservation analysis, and liver histology. **A** Family with *ABCC2* (c.2414delG, p.(Ile1489Gly), p.(Thr1490Pro), and p.(Ile1491Gln)) and *UGT1A1* mutations (c.-3279T>G, p.(Gly71Arg), and p.(Pro451Leu)). The proband is marked by arrow; black symbols denote affected members; white symbols denote unaffected members; squares denote men; and circles denote women. The sequencing chromatogram is shown. **B** Sequence alignment of *ABCC2* and *UGT1A1* is conserved among vertebrates (box). **C** Liver histology of the proband and his brother. C1 The proband, II:3. Hematoxylin-eosin (H-E) staining. Brown granular deposits of lipofuscin are evident. C2 The proband’s brother, II:9. Scale bar, 20 μm

All family members had normal serum values of total protein, albumin, globulins, aspartate aminotransferase (AST), and alanine aminotransferase (ALT) (Table 1). Three of the 11 members had been previously diagnosed with suspected hereditary hyperbilirubinemia (Fig. 1 and Table 1); all three had hyperbilirubinemia after birth, with no history of drinking, smoking, or exposure to special drugs/poisons or abnormal BMI, but the proband’s brother (II:9) had a long-term medication. The proband (II:3) and his brother (II:9) had unconjugated and conjugated hyperbilirubinemia, while the proband’s sister (II:1) had only conjugated hyperbilirubinemia.

**Table 1:** Clinical characteristics of the Chinese family with GS and DJS

Subject	TB (μmol/L)	DB (μmol/L)	IDB (μmol/L)	TP (mg/mL)	ALB (mg/mL)	GLB (mg/mL)	AST (IU/mL)	ALT (IU/mL)
I:1	6.4	3.5	2.9	69.4	35.1	34.3	29	31
I:2	8.6	3	5.6	78.1	42.1	36	18	16
II:1	**30.8**	**24.5**	6.3	76.7	47.1	29.6	19	34
II:2	5.2	4	1.2	67.5	42.6	24.9	21	8
II:3	**164**	**133**	**31**	49.9	29.3	20.6	30	27
II:4	7.8	4.8	3	72.3	46.1	26.2	26	19
II:5	13.7	3.9	9.8	69	38.8	30.2	16	13
II:6	8.8	5.2	3.6	64.6	45.2	19.4	37	32
II:7	7	1.9	5.1	77.4	41.9	35.5	13	13
II:9	**63.5**	**45.8**	**17.7**	77.6	44.6	33	22	30
II:10	9	3.7	5.3	70.9	46	24.9	15	11
III:1	7	4	3	70.3	46.1	24.2	31	13
III:2	8.8	2.7	6.1	74.1	48.5	25.6	17	26
III:3	7.3	2.8	4.5	67.4	40.3	27.1	21	28
III:4	6.5	2.5	4	76.6	43.9	32.7	17	21
III:5	9.9	3.6	6.3	74	45.5	28.5	15	16

Bold indicates deviation from normal values

*TB* total bilirubin (normal values: 3.0–21.0 μmol/L), *DB* direct bilirubin (normal values: 0–7.0 μmol/L), *IDB* indirect bilirubin (normal values: 1.7–14.0 μmol/L), *TP* total protein (normal values: 60.0–83.0 mg/mL), *ALB* albumin (normal values: 34.0–54.0 mg/mL), *GLB* globulin (normal values: 15.0–35.0 mg/mL), *AST* aspartate aminotransferase (normal values: 8.0–40.0 IU/mL), *ALT* alanine aminotransferase (normal values: 5.0–40.0 IU/mL)

The male proband (II:3) had cutaneous and scleral icterus at birth without a known cause. At age 50, he developed into pleural effusions, ascites, pericardial thickening, intrahepatic and extrahepatic biliary duct dilatation, and an enlarged gallbladder seen on computed tomography scan. Liver ultrasonography demonstrated normal liver size and morphology and uniform density. A mediastinal lymph node biopsy revealed reactive hyperplasia. Serologic tests were negative for Epstein–Barr virus, cytomegalovirus, and hepatitis A, B, C, D, or E viruses. He had no hematologic disorder, skin rash, or hepatosplenomegaly. Serum values of AST, ALT, α1-antitrypsin, copper, ceruloplasmin, thyroid hormones, sweat chloride, and screening biochemistries for metabolic diseases were within the normal range. However, severe hyperbilirubinemia was detected: total bilirubin 164 µmol/L (normal: 3.0–21.0 µmol/L) and direct bilirubin 133 µmol/L (normal: 0–7.0 µmol/L). In all, 2+ urinary bilirubin and 1+ urobilinogen were also recorded. Gamma-glutamyl transpeptidase activity was 136 U/L (normal: 11.0–50.0 U/L). After treatment, the total bilirubin stabilized at 80 µmol/L.

The proband’s brother (II:9) also had cutaneous and scleral icterus at birth without a known cause. At age 43, he had chronic cholecystitis and cholelithiasis. He took Xiaoyan Lidan tablets (Chinese patent medicine) regularly and amlodipine and metoprolol to control blood pressure. He developed cirrhosis and primary hepatocellular carcinoma at age 48 and died within 5 months of the emergence of the liver cancer. Hepatitis B infection was diagnosed at age 18, but HBeAb and HBcAb tests were positive without HBV DNA, suggesting spontaneous resolution of the hepatitis B infection. Serological tests for other hepatotropic viruses (hepatitis A, C, and D; Epstein–Barr virus; and cytomegalovirus) were negative. No hematologic disorders, autoimmune liver diseases, abnormal ceruloplasmin or serum ferritin, skin rash, or hepatosplenomegaly were detected. Mild hyperbilirubinemia (total bilirubin 63.5 µmol/L, direct bilirubin 45.8 µmol/L) at age 46 increased to severe hyperbilirubinemia (total bilirubin 371.4 µmol/L, direct bilirubin 286.1 µmol/L) with development of the liver cancer. Hepatocellular carcinoma was diagnosed by liver biopsy (Supplementary Fig. S2).

The proband’s sister (II:1) had conjugated hyperbilirubinemia (total bilirubin 30.8 µmol/L, direct bilirubin 24.5 µmol/L) without known disease or other causes of persisting hyperbilirubinemia (Table 1).

### Detection of *UGT1A1* and *ABCC2* mutations

The pedigree analysis of the family was conducted (Fig. 1). The genotypes of all family members were consistent. Genetic analysis of the proband revealed two mutations in *ABCC2*: a heterozygous deletion of c.2414delG in exon 18, inherited from his mother, and three heterozygous mutations [p.(Ile1489Gly), p.(Thr1490Pro), and p.(Ile1491Gln)] in exon 31, inherited from his father (Fig. 1 and Table 2). An evolutionary conservation analysis revealed that mutations p.(Ile1489Gly), p.(Thr1490Pro), and p.(Ile1491Gln) led to a highly conserved amino acid change (Fig. 1). Two disease-causing mutations were found by MutationTaster (https://www.mutationtaster.org/), but they were not observed in the SNP database (https://www.ncbi.nlm.nih.gov/snp/). The alterations were not identified in 211 unrelated control subjects from the same southwest Chinese population (data not shown) or in the 1000 Genome Project dataset (http://browser.1000genomes.org/index.html).

**Table 2:** Mutation status of the Chinese family with GS and DJS

Subject	Gender	Age (years)	Genomic mutations		CDS mutations of *ABCC2*
			*UGT1A1*	*ABCC2*	
I:1	Male	74	c.-3279T>K, p.(Pro451Leu)	p.(Ile1489Gly), p.(Thr1490Pro), p.(Ile1491Gln)	p.(Ile1489Gly), p.(Thr1490Pro), p.(Ile1491Gln)
I:2	Female	72	c.-3279T>K, p.(Gly71Arg), p.(Pro364Leu)	c.2414delG	NA
II:1	Female	51	c.-3279T>K, p.(Pro364Leu)	c.2414delG, p.(Ile1489Gly), p.(Thr1490Pro), p.(Ile1491Gln)	c.2414delG, p.(Ile1489Gly), p.(Thr1490Pro), p.(Ile1491Gln)
II:2	Male	54	NA	NA	NA
II:3	Male	54	c.-3279T>K, p.(Gly71Arg), p.(Pro451Leu)	c.2414delG, p.(Ile1489Gly), p.(Thr1490Pro), p.(Ile1491Gln)	c.2414delG, p.(Ile1489Gly), p.(Thr1490Pro), p.(Ile1491Gln)
II:4	Female	54	–	–	–
II:5	Female	44	c.-3279T>K, p.(Pro364Leu)	p.(Ile1489Gly), p.(Thr1490Pro), p.(Ile1491Gln)	p.(Ile1489Gly), p.(Thr1490Pro), p.(Ile1491Gln)
II:6	Male	45	NA	NA	NA
II:7	Female	40	p.(Gly71Arg)	c.2414delG	c.2414delG
II:9	Male	46	c.-3279T>K, p.(Gly71Arg), p.(Pro451Leu)	c.2414delG, p.(Ile1489Gly), p.(Thr1490Pro), p.(Ile1491Gln)	c.2414delG, p.(Ile1489Gly), p.(Thr1490Pro), p.(Ile1491Gln)
II:10	Female	42	NA	NA	NA
III:1	Male	27	p.(Gly71Arg)	p.(Ile1489Gly), p.(Thr1490Pro), p.(Ile1491Gln)	p.(Ile1489Gly), p.(Thr1490Pro), p.(Ile1491Gln)
III:2	Male	29	p.(Gly71Arg)	c.2414delG	NA
III:3	Female	30	c.-3279T>K, p.(Pro451Leu)	p.(Ile1489Gly), p.(Thr1490Pro), p.(Ile1491Gln)	p.(Ile1489Gly), p.(Thr1490Pro), p.(Ile1491Gln)
III:4	Male	17	p.(Pro364Leu)	–	NA
III:5	Female	20	NA	NA	NA

“–” no mutation was detected, *NA* not available

For the *UGT1A1* gene, the proband had a polymorphic mutation c.-3279T>G in the promoter, a heterozygous mutation p.(Gly71Arg) in exon 1, and another heterozygous mutation, p.(Pro451Leu), in exon 5 (Fig. 1 and Table 2). Mutation p.(Pro451Leu) is an evolutionary conserved amino acid change (Fig. 1). Other family members were also investigated for defects in the *ABCC2* and *UGT1A1* genes. The proband and his brother (II:9) had the same genetic defect in *ABCC2* and *UGT1A1*. The proband and his sister (II:1) had similar mutations in the *ABCC2* gene. Four members of the family were found to have another mutation in *UGT1A1* [p.(Pro364Leu)], which was absent in the proband (Table 2).

### Verification of *ABCC2* mutations in CDS

To investigate the transcriptional mutation of the *ABCC2* mutations in our samples, exon 18 and exon 31 were amplified from cDNA of all the family-based samples. The heterozygous deletion mutation c.2414delG in exon 18 of II:1, II:3, II:7, and II:9 were the same as that in the genomic DNA but were not available in I:2 and III:2 (Table 2). We got two PCR bands of exon 31 in cDNA from samples who carry those mutations in genomic DNA, but only one band in wild-type controls (Supplementary Fig. S1A). One of the 2 bands covered the full length of exon 31 with the three consecutive heterozygous mutations [p.(Ile1489Gly), p.(Thr1490Pro), and p.(Ile1491Gln)], and the other band has a 195-bp deletion (c.4314-4508del) (Supplementary Fig. S1B). The three consecutive heterozygous mutations were verified by TA cloning (Supplementary Fig. S1C). Sequencing of the full-length CDS of the proband showed that the heterozygous deletion mutation c.2414delG in exon 18 do not occur in the full-length 4638-bp CDS of the *ABCC2* gene. While, the three consecutive mutations were homozygous in the full-length 4638-bp CDS of the proband (Supplementary Fig. S1D), which were different from that of genomic DNA.

### Histology and ABCC2 protein expression

For the proband, liver histology revealed normal structure and intense, brown parenchymal deposits of lipofuscin with a centrilobular and midzonal accentuation in liver cells (Fig. 1C). The pigment was strongly PAS positive (Supplementary Fig. S3) but was negative in Masson’s reaction. The accumulation of pigment was suggestive of that seen in DJS [8], but with distinctive brown granules. Perl Prussian blue staining for hemosiderin was negative, indicating normal hepatic iron load. The proband’s brother (II:9) had liver histopathological characteristics like those of the proband (Fig. 1C2 and Supplementary Fig. S3). Supplementary Fig. S4 illustrates that the lipofuscin pigment in the hepatocytes of the proband’s brother (II:9) was present in hepatocytes adjacent to cancer cells (Supplementary Fig. S4A) but not in the cancer cells (Supplementary Fig. S4B).

**Fig. 2:**
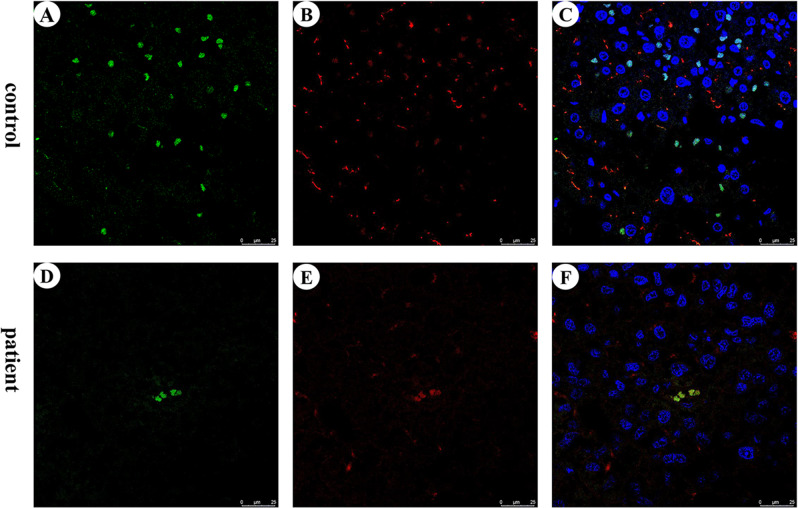
Localization of ABCC2 (**A** and **D**, green) and CEACAM1 (**B** and **E**, red) by immunofluorescence staining in liver tissues of control and proband. Cell nuclei are stained with 4’, 6-diamidino-2-phenylindole (DAPI) (**C** and **F**, blue). Hepatocytes positive for CEACAM1 as well as ABCC2 are more numerous in control tissue than in the proband’s tissues. Cells positive for the target proteins and the cell nuclei are stained in the same sections (**C** and **F**). Scale bar, 25 μm

ABCC2 protein expression was analyzed with CLSM double immunofluorescence staining. In the normal liver, ABCC2 and CEACAM1 had similarly high expression (Fig. 2A–C), but in the patient’s liver, the expression of ABCC2 was lower than that in the control group (Fig. 2D–F).

## Discussion

The concurrence of mutation characteristics of DJS and GS has been designated dual-hereditary jaundice [9, 10]. Herein, we report a compound defect of *ABCC2* [c.2414delG, p.(Ile1489Gly), p.(Thr1490Pro) and p.(Ile1491Gln)] and *UGT1A1* genes [c.-3279T>G, p.(Gly71Arg) and p.(Pro451Leu)] causing DJS and GS in a Han Chinese family, which are not exactly the same mutations as identified in reported dual-hereditary jaundice patients [7, 9, 10]. Although many genetic alterations of the *ABCC2* gene have been identified in DJS patients, hotspot mutations are not among them [11]. The new variant, c.2414delG, is in the exon 18 of the *ABCC2* gene. The deletion leads to frameshift and truncation of the encoded ABCC2 protein from the original 1545 amino acids to 809 residues. The truncated protein damages three ATP binding cassettes and three transmembrane domains and probably degrades newly formed mRNA rapidly [12]. In this family, we also found three continuous coding variants [p.(Ile1489Gly), p.(Thr1490Pro), and p.(Ile1491Gln)] in exon 31 of *ABCC2*, which is a rare mutational profile. p.(Ile1489Gly), p.(Thr1490Pro), and p.(Ile1491Gln) have been predicted to be “probably damaging” to protein function and structure, with scores of 0.996, 1, and 1 base on PolyPhen-2 (Harvard Medical School, Boston, Massachusetts, USA), which had been reported [9]. We also found diminished expression of ABCC2 in the brother of the proband (II:9) with indel and mutations, a finding that may indicate that defective *ABCC2* caused impaired protein function. Furthermore, indel and mutations were co-segregated with the phenotype of congenital conjugated hyperbilirubinemia for family members II:3, II:9, and II:1, which suggests that this mutation severely disrupts its transporter activity. However, the transcriptional status may be different between the family members who harbor the same mutation(s) in the genomic DNA according to the CDS sequences. The different transcriptional results may have a role in the different disease phenotype among the family members who share the same genomic DNA mutation(s). Unfortunately, we failed to acquire the full-length CDS of the *ABCC2* gene of the other family members. Defective ABCC2 is responsible for DJS with melanin-like pigment in the hepatocytes. Unlike this black pigment, the liver of DJS has deposits of brown lipopigment, which are consistent with the results of Cebecauerova et al. [10] in a dual-hereditary jaundice case. The pigment in the hepatocytes of our proband’s brother was present in hepatocytes adjacent to cancer cells but not in the cancer cells, suggesting that liver cancer cells have a different metabolic mechanism for handling pigment deposits or lipofuscin. More evidence is needed to explain these findings.

Three coding variants [p.(Gly71Arg), p.(Pro364Leu), and p.(Pro451Leu)] and one promoter variant c.-3279T>G were identified in the *UGT1A1* gene. Sense polymorphic mutations, A(TA)_7_TAA and c.-3279T>G in *UGT1A1*, were most strongly associated with GS, whereas mutations p.(Gly71Arg) and p.(Tyr486Asp) were more strongly associated with CNS-II [6]. Thus, we identified a different mutation profile with GS. Three variants [c.-3279T>G, p.(Gly71Arg), and p.(Pro451Leu)] were co-segregated with the phenotype of congenital unconjugated hyperbilirubinemia, which may account for GS. c.-3279T>G, p.(Gly71Arg), and p.(Pro364Leu) have been reported to contribute to GS, with bilirubin UGT activity reduced to 62%, 40%, and 36.6% of normal, respectively [13]. Though the role of p.(Pro451Leu) in UGT1A1 isoenzyme activity has not been reported, sorting intolerant from tolerant and polymorphism phenotyping analyses [14] and the correlation of phenotype-genotype observed in our pedigrees make it persuasive that this mutation was the cause of unconjugated hyperbilirubinemia. Although some studies suggest that compound variants [c.-3279T>G, p.(Gly71Arg), and p.(Pro364Leu)] make UGT1A1 protein failure, some family members (I:2, II:1, II:9, and III:4) who had these mutations had normal unconjugated bilirubin values. This finding suggests that the mutations have variable phenotypes among populations. We speculate that because of some compensatory mechanism in the family described herein, down-regulated UGT1A1 isoenzyme activity was not consistent with unconjugated hyperbilirubinemia.

Dual-hereditary jaundice has been described infrequently [7, 10], and we are aware of the lack of study which has followed such patients from infancy. Here, we found that the proband and his brother, who have the same defects in two genes and similar unconjugated and conjugated hyperbilirubinemia, developed different diseases in a span of 50 years. Bilirubin has two clinical behaviors: mild hyperbilirubinemia has health-promoting effects, which can protect from type 2 diabetes mellitus, cardiovascular diseases, and some cancers [15, 16]. However, severe hyperbilirubinemia can have adverse outcomes, such as jaundice, neurotoxicity, and cholestasis [17]. Few studies have explored the clinical outcomes of unconjugated and conjugated hyperbilirubinemia in GS and DJS. The proband in our study had 50 years of dual jaundice yet did not develop into severe liver-related disease. The proband’s brother, who had a long medication history, hypertension, and cholelithiasis, developed into hepatocellular carcinoma at age 48. So far, at least three studies had suggested that DJS coexisted with HCC [18–20]. The medication history of the two patients was compared; the proband’s brother had long-term medication for liver diseases, while the proband had no long medication history. Because UGT1A1 and ABCC2 proteins are responsible for drug biotransformation, defects in them may impair their ability to metabolize certain drugs and increase their toxicity. More similar cases or function verification may reinforce the conclusion.

In conclusion, we describe a Chinese family with two brothers who had combined GS and DJS. Compound defects of the *ABCC2* gene [c.2414delG, p.(Ile1489Gly), p.(Thr1490Pro), and p.(Ile1491Gln)] and the *UGT1A* gene [c.-3279T>G, p.(Gly71Arg), and p.(Pro451Leu)] were identified. One brother developed hepatocellular carcinoma after long-term treatment with multiple medications. The present study illuminates the underpinnings of GS and DJS, and extends mutation profiles and phenotypes of the syndromes.

## Supplementary information

Supplementary material

Supplementary material
