# Supplementary material for: Concurrence of novel mutations causing Gilbert’s and Dubin–Johnson syndrome with poor clinical outcomes in a Han Chinese family
Source: J Hum Genet. 2022 Oct 24;68(1):17–23. doi: 10.1038/s10038-022-01086-1 (PMC9812767; doi:10.1038/s10038-022-01086-1)
Supplement: Supplementary file 1 — Supplementary material [file 10038_2022_1086_MOESM1_ESM.pdf]

Supplementary Figures

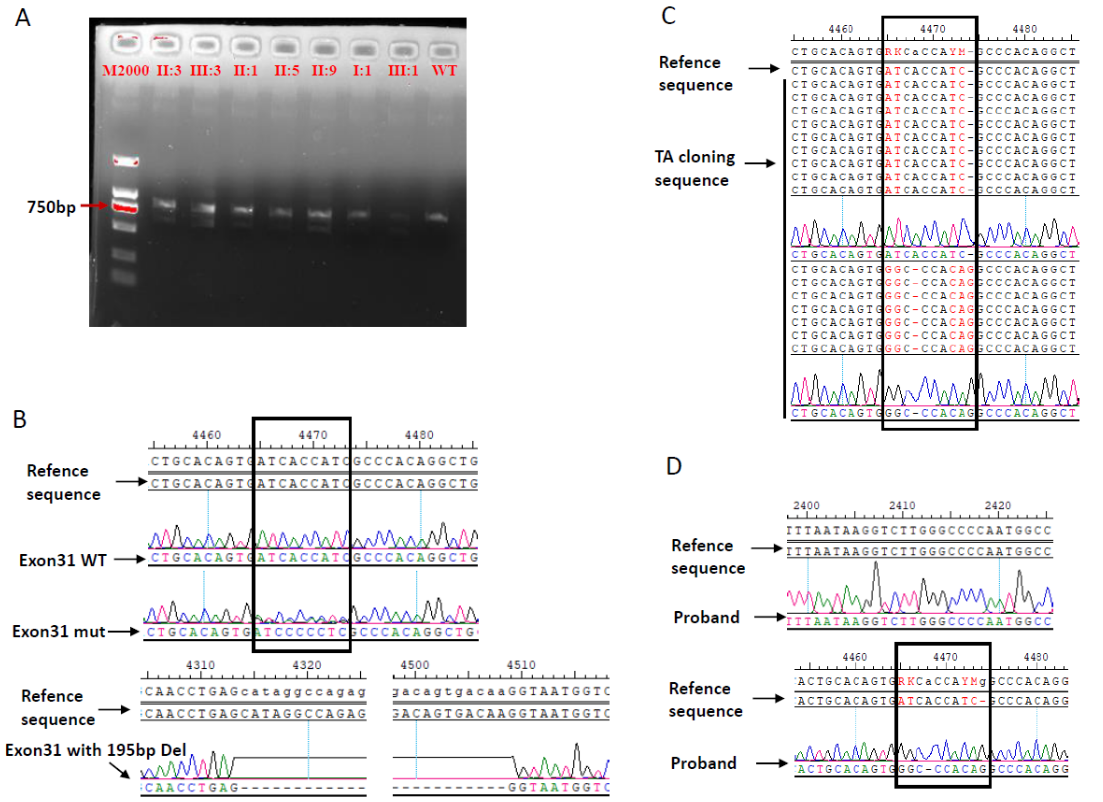

Supplementary Figure 1. Verification of the genomic DNA mutations in cDNA. (A) PCR products of exon 31 from cDNA. We amplified the 7 individuals who has the continuous coding variants [p.(Ile1489Gly), p.(Thr1490Pro), and p.(Ile1491Gln)]in exon 31 of the *ABCC2* gene and a wild type control from their family. M2000, DNA marker 2000. (B) Sequencing electropherograms of exon31. Due to the space limitation, we present one electropherogram referring to each mutation status. (C) TA cloning and sequencing to verify the heterozygous mutation p.(Ile1489Gly), p.(Thr1490Pro), and p.(Ile1491Gln) of exon 31 of the *ABCC2* gene. (D) Sequences cover c.2414 in exon 18 and c.4465-4473 in exon 31 from full length CDS of the *ABCC2* gene of the proband.

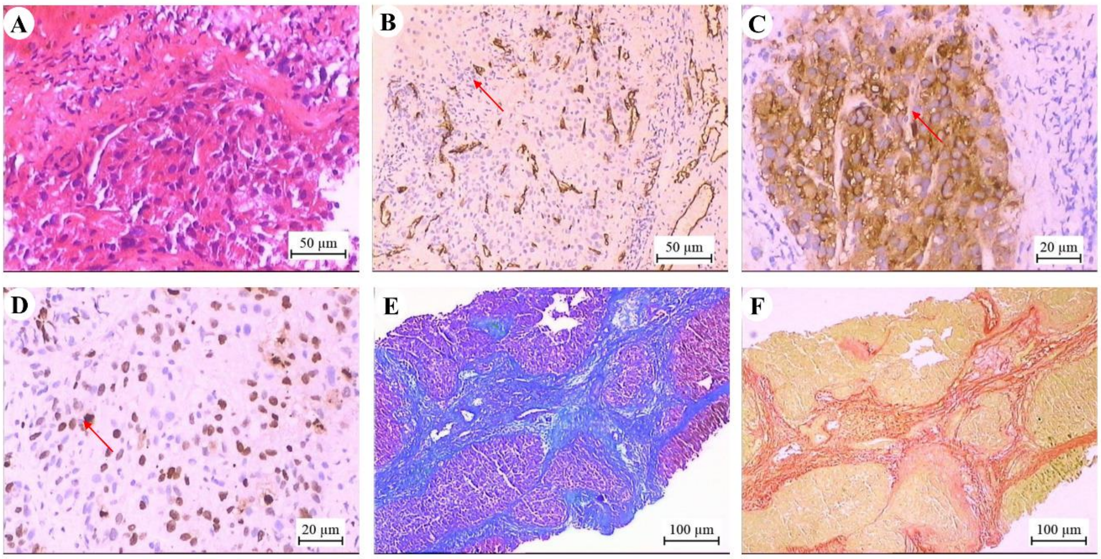

Supplementary Figure 2. Liver histopathology of the brother of the proband (II:9). (A) H-E staining indicates cholestasis in hepatocytes, proliferation of peripheral fibrous tissue, and chronic inflammatory cell infiltration. (B) Immunohistochemical staining of CD34 (positive) indicates hepatic sinus fibrosis. (C) Immunohistochemical staining of glypican (positive). (D) Immunohistochemical staining of KI-67 (positive). (E) Masson staining indicates proliferation of fibrous tissue. (F) Silver staining indicates proliferation of fibrous tissue.

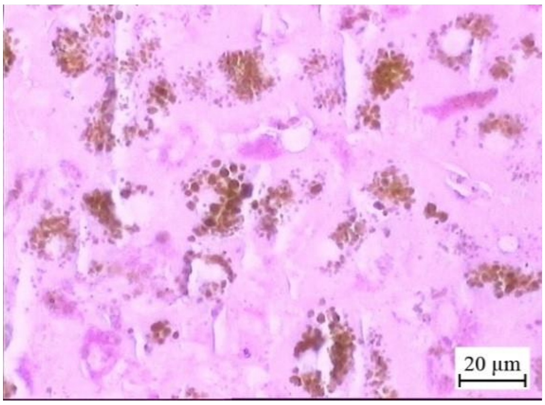

Supplementary Figure 3. Proband's brother (II:9). PAS-stained liver histology. (Original magnification_400).

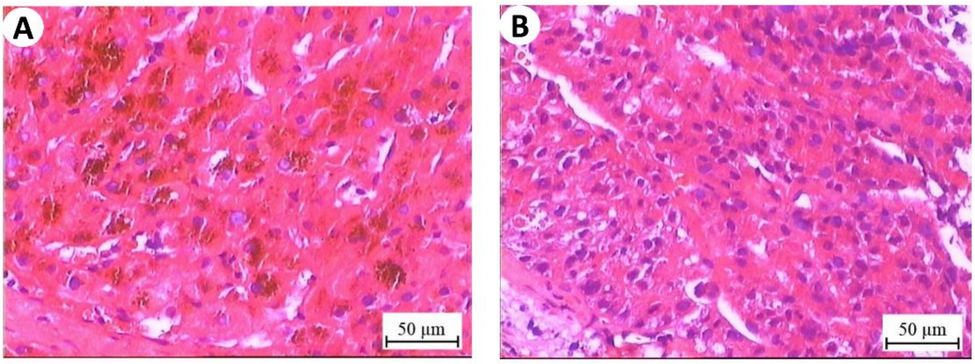

Supplementary Figure 4. Liver histology of the proband's brother (II:9), which indicates that liver para-carcinoma tissue stores brown granular deposits (A) but liver cancer tissue does not (B). (Original magnification_200).
