# Supplementary material for: Concurrence of novel mutations causing Gilbert’s and Dubin–Johnson syndrome with poor clinical outcomes in a Han Chinese family
Source: J Hum Genet. 2022 Oct 24;68(1):17–23. doi: 10.1038/s10038-022-01086-1 (PMC9812767; doi:10.1038/s10038-022-01086-1)
Supplement: Supplementary file 2 — Supplementary material [file 10038_2022_1086_MOESM2_ESM.docx]

**Supplemental materials**

**Table S1.** The primers used for CDS amplification and sequencing of *ABCC2* gene.

| primers | sequence | applications |
| --- | --- | --- |
| **For exon18** | | |
| ABCC2-e18-F | TGTCCACGGGCACATCAC | Exon 18 amplification and sequencing |
| ABCC2-e18-R | TTGGACCTAGAACTGCGGCTAA | Exon 18 amplification and sequencing |
| **For exon31** | | |
| ABCC2-e31-F | AACTGCCTCTTCAGAATCTTAG | Exon 31 amplification and sequencing |
| ABCC2-e31-R | GGTGGGTAGCAAGGAAGTACGAT | Exon 31 amplification and sequencing |
| **For full length CDS** | | |
| ABCC2-90F | TCTTCGTTCCAGACGCAGTC | Full length CDS amplification and sequencing |
| ABCC2-4977R | GGGTGGGTAGCAAGGAAGTAC | Full length CDS amplification and sequencing |
| ABCC2-cds-489R | GGCTAGATTAGAATTGTCACCCTGT | Full length CDS sequencing |
| ABCC2-cds-1431F | TGCGATACTGTCCACCAAGAG | Full length CDS sequencing |
| ABCC2-cds-2071F | GTCCACGGGCACATCACCA | Full length CDS sequencing |
| ABCC2-cds-2737F | CTTAGCCGCAGTTCTAGGTCC | Full length CDS sequencing |
| ABCC2-cds-3277F | GACACCCTGCCTCAGTCCTTG | Full length CDS sequencing |
| ABCC2-cds-3966F | TGACATCGGTAGCATGGAGAAG | Full length CDS sequencing |

Note: CDS, coding sequence.
